# Recreation- and sport-led regeneration of urban water infrastructure

**DOI:** 10.3389/fspor.2025.1558415

**Published:** 2025-04-30

**Authors:** Karin Book

**Affiliations:** Department of Sport Sciences, Malmö University, Malmö, Sweden

**Keywords:** sport, recreation, regeneration, harbour, canal, ecosystem services, sustainability, tourism

## Abstract

Today, cities are to a growing extent looking for solutions for how urban infrastructure, like former industrial sites, can be developed into facilities for sport and recreation, as well as tourism. One example of such infrastructure is canals and former harbour areas. This paper aims at exploring the underlying factors behind, and the potential benefits and challenges of, recreation- and sport-led regeneration of urban water infrastructure with a focus on former harbours and canals, using three Scandinavian cities as examples: Copenhagen, Gothenburg and Malmö. A conceptual and theoretical frame is built around three perspectives: (1) contemporary trends and tendencies in sport and recreation, and spatial implications thereof, (2) urban regeneration, and (3) recreational spaces as ecosystem services. The empirical material is mainly based on six semi-structured interviews with informants involved in the planning, production and operation of the water infrastructure. As shown by the three examples, there are several benefits of a recreation- and sport-led regeneration of former harbours and canals. Those benefits include, for instance, ecosystem services, such as enhanced biodiversity and improved recreational opportunities, quality of life and well-being, as well as economic benefits in terms of tourism and positive attention. One of the examples also demonstrates that harbour regeneration could be an opportunity to develop methods for community participation and public-nonprofit partnerships. However, the examples highlight a number of challenges as well, such as polluted water and the technical issues and high costs involved in cleaning it; the provision of equal access to the water infrastructure; the creation of a safe co-existence for different actors and activities in the same water space; and diverse ownership, responsibility and regulation issues. To summarize, the paper shows that in a successful recreation-led regeneration of urban water infrastructure, the potential outcome is increased attractivity, activity and sustainability.

## 1 Introduction

One of the features gaining most attention during the Olympic Games in Paris 2024 was the river Seine as an arena for both the opening ceremony and various swimming events (including triathlon). It was an exciting but very problematic and costly strategy. Making the river sufficiently clean is said to have cost around one and a half billion euros. Among other things, new, large water reservoirs were required to ensure that sewage did not come out into the Seine in the event of heavy rains. And it certainly did rain during the opening of the Games. The criticism from the athletes who were forced to swim in the Seine was not insignificant. Thus, the potential opportunity to market the venture as both spectacular and sustainable was reduced. However, if this investment in the long run has positive effects on the environment and gives Parisians new recreational opportunities, in addition to the spectacular exposure during the Olympics itself, then it can probably be seen as a positive legacy.

The Seine example showcases how cities today are thinking in new ways regarding which infrastructural resources can be used as facilities for sport and recreation. This applies not least to canals and harbour areas, but also to industrial premises of various kinds. In Sweden, for instance, there is an ongoing discussion about the lack of facilities for sport. Maybe new solutions for, and new interpretations of, what a sport facility could be, are a way forward to create more opportunities for physical activity, as well as making the city more attractive. Moreover, the Seine example highlights the branding potential of urban recreation solutions, as well as the environmental challenges connected to them.

In an article in the New York Times, Ayuso (1, no page), presents the urban trend to develop “blue infrastructure”, like swimming pools in rivers and “harbour baths”, and how this can “help cool cities, promote biodiversity and improve quality of life”. There are, it would seem, environmental and socio-economic as well as economic perspectives linked to the development of blue infrastructure.

This paper aims at exploring the underlying factors behind, and the potential benefits and challenges of, recreation- and sport-led regeneration of urban water infrastructure with a focus on former harbours and canals. On the one hand, the paper builds on three Scandinavian examples: Copenhagen, Gothenburg and Malmö. On the other hand, it has a conceptual and theoretical frame built around three perspectives, functioning as a starting point for understanding the recreation-led regeneration of urban water infrastructure. Firstly, urban regeneration as a concept is briefly discussed. Secondly, contemporary trends and tendencies in sport and recreation, and spatial implications thereof, are discussed. Thirdly, the perspective of recreational spaces as ecosystem services is introduced.

## 2 Conceptual frame

The amount of research on the development of water, in terms of urban harbours and canals, for recreational purposes remains limited. However, if we include literature on the related phenomenon of waterfront development, there is a vast body of work from the late 1980s to the present. While waterfront development is not the precise focus of this paper, it could serve as a valid starting point. In the last 30–45 years, our attention has increasingly turned towards the waterfront areas of port cities, although “the interlinkages between the port function and urban form have provided interdependencies throughout the history of city development” (2, p. 397).

In the 1980s and 1990s, deindustrialization in port cities across the Western world led to a surge in waterfront (re)development, with London Docklands serving as a notable example. Much of the academic literature links waterfront development to urban entrepreneurialism and neoliberal approaches to urban economic growth (e.g., 3, 4), involving “local boosterism, place marketing of cities, and high demands on the management of funding and financial risks” (5, p. 266). This has often resulted in large-scale flagship initiatives driven by public-private partnerships (6). These projects frequently feature high-value housing and iconic architecture with a strong commercial focus, leading to consequences such as the privatization of space, gentrification, and exclusion (see e.g., 7, 8). Tommarchi (9) emphasizes the consumption aspect, noting that “waterfront redevelopment has often been about replacing port/industrial functions with tertiary functions or spaces for consumption” (p. 12). Additionally, Tommarchi highlights that in the 1990s major or mega events often triggered these large-scale redevelopments, referencing Andrade and Costa's (10) concept of the “mega-tertiary waterfront.” Waterfront development is often described based on certain features, originating from the 1980s and 1990s. However, Tommarchi identifies slightly different periodisations (typologies) of waterfront redevelopment, in terms of dominant approaches guiding the transformation of the waterfront. From the 2000s and onwards two different approaches are identified. One is based on late neoliberal approaches with a focus on maximizing the value that can be extracted from urban spaces and assets. The other includes holistic approaches with more balanced strategies focusing on various dimensions of sustainability. According to Tommarchi (9):

[…] holistic approaches to waterfront redevelopment are also expected to become more widespread as a means to address climate change adaptation in coastal or riverside port cities (through coastal defence and water management infrastructure, mitigation of urban heat island effects, and carbon sequestration), and to pursue sustainable development more broadly in areas such as liveability, wellbeing, cultural opportunities, and inclusion (pp. 11–12).

Hermelin and Jonsson (5) identify a similar change:

While strongly commercially driven approaches were identified for projects developed during the 1980s and 1990s, more recent projects have been found to take broader perspectives with respect to social aims and concerns about environmental sustainability (11) (p. 266).

Sairinen and Kumpulainen (12) also emphasize the social dimensions of urban waterfront regeneration:

[…] considering the social impacts and aspects of waterfront regeneration has become increasingly important task for both the legitimacy and the actual substance development of waterfront projects and plans (p. 122).

This paper focuses on urban water infrastructure, which may or may not be directly linked to waterfront (re)development. The regeneration of urban canals and harbours can perhaps be seen as an extension of waterfront development into the water, particularly in relation to the holistic waterfront approach (see 9).

This paper should be considered an exploratory and conceptual work, aiming to frame a phenomenon that is becoming increasingly common in cities across Europe and other parts of the world. The goal is not to present a comprehensive and systematic literature review, but rather to construct a framework based on the three perspectives mentioned in the introduction: urban regeneration; contemporary trends and tendencies in sport and recreation; and recreational spaces as ecosystem services.

Linking to the discussion on waterfront development above, the first perspective to be explored is urban regeneration.

### 2.1 Urban regeneration and the move towards community orientation

During the late 1970s and the 1980s, a considerable number of the former industrial cities in the western world, of which Malmö, Gothenburg and Copenhagen are good examples, experienced an economic restructuring. Deindustrialisation was at the core of this change and, as a consequence, many cities lost their main economic base, and former industrial infrastructure, such as factories, warehouses and harbour areas, were deprived of its functions (e.g., 13, 14). Hence, the former industrial cities had to promote and develop favourable conditions for other economic activities, often with a focus on service-, knowledge- and information-based industries, but also on experience economy and tourism (15, 16). As part of the transformation, urban redevelopment and regeneration strategies based on sport and leisure have emerged as a critical feature of the post-modern city of consumption, with sport events, sport infrastructure and sport programmes being important in facilitating the transformation (16, 17). Research on this has mainly focused on urban regeneration in relation to sport events (17, 18); see also the above section on waterfront development).

Davies (17) discusses the meaning of urban regeneration, which could, according to her, include aspects of place marketing and property speculation, the physical redevelopment and reconstruction of an area (e.g., a waterfront), as well as the economic, social and environmental transformation of urban areas, or the long-term, lasting transformation of an area that has previously suffered some sort of degeneration. Despite underlining the evolving nature of urban regeneration, Roberts (19) presents a definition that is similar to Davies' account of the concept. He defines it as “comprehensive and integrated vision and action which leads to the resolution of urban problems and which seeks to bring about a lasting improvement in the economic, physical, social and environmental condition of an area that has been subject to change or offers opportunities for improvement” (p. 18). Also, Roberts (19) argues that “[u]rban regeneration moves beyond the aims, aspirations and achievements of urban renewal, which is seen […] as ‘a process of essentially physical change’ [with a reference to (20)]” and also beyond urban “redevelopment” and urban “revitalisation” (p. 18).

Davies (17) adds sport to the concept and discusses the meaning of sport-related urban regeneration, one useful definition being: “the way that sport can be used to revitalize an area economically, socially, environmentally and physically” (p. 1539). In her article on sport urbanism, Lioce (21), no page) elaborates on the fact that sport urbanism is “increasingly recognized as a crucial strategy in the regeneration of cities”, arguing that “[t]his approach advocates for integrating sports into urban design as a green, inclusive solution that enhances the beauty and functionality of public spaces”, and that it “involves designing urban spaces that prioritize physical activity, community interaction, and environmental sustainability”. She emphasises that “[b]y integrating sports facilities, parks, and green corridors into urban areas, cities can create environments that promote health, well-being, and social cohesion”.

I would like to argue that a shift in sport-led urban regeneration has occurred: from mainly using sport as a means to create regeneration with a focus on place branding and attracting tourists, towards a more community-oriented approach with the ambition to create both branding effects and attractive, functional opportunities for the residents (see also 22, 23). This ties in with Book and Svanborg Edén (15), according to whom “[i]t has […] become more common to embrace and highlight other aspects of sport than the spectacular when developing and marketing the city, like health aspects, innovative outdoor activity spaces, bike-friendliness or skate-friendliness” (p. 169). This could also be linked to contemporary sport and recreation trends.

### 2.2 Sport and recreation trends—spatial implications in an urban context

In the Scandinavian countries, as in several other countries, the urban landscape for physical activity seems to be changing, in both spatial and organisational terms. We can see a growing trend of moving away from traditional, formal, organised club sport towards self-organised activities in informal settings of different kinds (for a discussion on institutional changes, see, e.g., 24). It is worth underlining that, among active adults, self-organised sport and recreation activities are the most common (e.g., 25). Swedish statistics (idrottsstatistik.se) show that the ten most popular sport and exercise activities among the population (6–80 years old) in 2023 were walking, strength training, running, cycling, hiking, swimming, group training, football, yoga and dancing. Those are, maybe with the exception of football, not typical club-sport activities, but rather activities that are more frequently carried out in a self-organised way. However, despite the fact that Scandinavians generally have a high average physical activity level (see, e.g., the Eurobarometer on Sport and Physical Activity, 26, 27), we are witnessing increasing inactivity rates, especially among young people, and segregated activity patterns based on, among other things, socio-economic factors (e.g., 28).

In relation to declining physical activity levels, the planning and provision of places and facilities for sport, recreation and physical activity have gained increased attention in both practice and research. To meet the preferences for self-organised activities, and to create accessible opportunities and encourage physical activity for those who are inactive, municipalities are showing an increased interest in investing in public, informal outdoor sport and recreational spaces—such as outdoor gyms, skate parks, small-scale ball pitches, running trails in public parks, bike infrastructure, etc. (29–31). The strategies to build public, informal outdoor sport and recreational spaces, as opposed to closed, traditional sport facilities, can also be related to a larger urban planning ambition to make the city more attractive and liveable.

Over the past 20 years, many studies have focused on physical activity in relation to the built environment, public space and urban morphology (e.g., 32, 33). Investing in an infrastructure for sport and recreation, be it traditional sport facilities or nature-based recreation opportunities, is one thing, making it accessible is another thing. Several studies have linked engagement in physical activity to tangible factors like physical accessibility to sport infrastructure and public space opportunities in terms of relative proximity and actual travel times/distances to sport facilities and places, although with inconsistent findings (e.g., 34–36). Furthermore, aspects such as safety, amenities, restrooms, maintenance, aesthetics, environmental comfort, walking paths, human scale, visual vision and landscape have all been found to be important attributes for supporting physical activity (e.g., 32, 37, 38). In a study of public, informal outdoor sport spaces, Book and Högdahl (31) found that the following aspects influence the perceived accessibility of the place: guiding physical structures and inclusive edges; a variety of activities and opportunities; social support and guidance; and norms, representation and the feeling of belonging.

In Sweden as well as other countries, there is a general opinion in the organised sport movement that there is a lack of sport facilities, and a need for many existing facilities to be upgraded, especially in metropolitan areas (e.g., 39, 40). In parallel, cities are undergoing densification processes, with the aim of conserving land resources and developing in a more sustainable way. Densification has consequences for if and how the need for sport facilities is handled in planning. Ultimately, traditional sport facilities do not appear to correspond to contemporary exercising trends that need efficient, sustainable and, sometimes, profit-driven land use.

Hence, there are changing physical activity patterns, as well as increasing inactivity, a perceived need for more facilities among the actors of the sport movement and, at the same time, a growing competition over urban space. Therefore, new spatial solutions for sport, physical activity and recreation have to be developed, for example, compact concepts; new artificial and technological solutions; a redefinition of what a sport facility could be; and coordination between different functions, activities and actors.

In urban settings, most sport and recreational facilities are artificially constructed. Several studies and papers have focused on the trend and the problems of artiﬁcially constructed landscapes, like indoor or other artificial facilities for climbing, downhill skiing and cross-country skiing (see, e.g., 41–43). In these facilities, activities previously carried out in natural environments are taken out of their natural contexts. Sandell and Öhman (44) discuss different sport landscape approaches, namely, what they call the active domination approach and the active adaptation approach. The former refers to landscapes that are being manufactured, structured and equipped for a certain activity, while the latter instead means that the activity is adapted to the features of the landscape and natural conditions. Another useful concept, introduced by Sandell (45), is decontextualisation, referring to the renegotiation of the context for several outdoor sports.

Finally, the increased emphasis on green spaces in the cities is worth mentioning. On the one hand, there is a need to bring the urban population closer to nature, but, on the other hand, there is also an increasing demand for outdoor urban spaces where people can exercise and engage in outdoor activities and thus change daily habits for a healthier lifestyle. So, parks and other nature-oriented spaces can be a support infrastructure for a more sustainable lifestyle (46, 47). As populations have rapidly concentrated into urban areas that are largely man-made and highly segregated from nature, a decline in accessible urban green spaces has occurred (48–50). Hence, Liu et al. (51) underline that it is crucial to ensure the provision of opportunities for natural-based recreation.

### 2.3 Recreational spaces as ecosystem services

Pinto et al. (52) state that “incorporating NbS [nature-based solutions] and GBI [green and blue infrastructure] in urban planning and design is imperative for creating liveable cities, providing good health and supporting the ecosystems” (p. 7), and that it contributes to the United Nations' Sustainable Development Goals. As noted above, opportunities for natural-based recreation in urban areas are important. It is well known that outdoor recreation improves urban residents' health. Urban growth significantly increases the pressure on remaining urban open spaces and negatively impacts green spaces, indirectly impeding outdoor recreation and, as a consequence, the quality of life and well-being of the residents (e.g., 53). Liu et al. (54) emphasise that recreation in nature-related settings benefits people by providing aesthetic experiences, enhancing people's physical and psychological health, and increasing social cohesion. Therefore, it can be considered a type of ecosystem services.

Ferreira, Vasconcelos and Ferreira (47) provide the following definition of ecosystem services: “the benefits obtained directly or indirectly from ecosystems, whether they are natural or naturalized or semi-natural, that is, the capacity of natural processes or components of ecosystems to provide goods and services that satisfy directly or indirectly human needs” (p. 1). Ecosystem services thus incorporate ecological, sociocultural (or social) and economic values.

The ecological, or environmental, ecosystem services have a number of benefits in that they, for instance, promote biodiversity; contribute to improved air quality; regulate air temperature; help mitigate the risk of flooding; and help reduce noise (47).

The social benefits include a positive impact in society and on individuals by providing a space for free and recreational activities. As mentioned above, this could be related to improving people's physical and psychological health and well-being. Vierikko and Yli-Pelkonen (55), for instance, emphasise, with references to the concept of ecosystem services, that urban water ecosystems are popular among urban residents to visit and important from a well-being perspective.

As for the economic benefits of the ecosystem service, they can, just like the ecological and social benefits, be at play at different levels. For instance, the ecosystem service can enhance the quality of surrounding areas and thereby increase the value of land and real estates. Also, the aesthetic, recreational and cultural functions of these spaces, can help promote tourism and the urban attractiveness and brand (56). Moreover, environmental improvements can have a positive economic effect for the city. The recreational and social values can, furthermore, be beneficial for mental and physical health, which is not only good from an individual but also from a societal perspective, due to, for instance, potentially reduced medical costs and increased productivity (47).

The economic effects of the ecosystem service are not necessarily purely positive, however. Pinto et al. (52) highlight that regeneration strategies are increasingly capitalising on greening initiatives, and that urban green and blue infrastructure can drive gentrification. As for increased property values, they could have both positive and negative effects. The latter include social exclusion and inequalities.

### 2.4 Summarising the conceptual framework

The above, partly over-lapping, perspectives form a conceptual framework for interpreting the growing phenomenon of recreation- and sport-led regeneration of urban water infrastructure illustrated in the figure below. They give rise to a number of questions, for instance:

How does the regeneration of urban water infrastructure relate to the trends in sport and physical activity, and not least to the spatial implications and challenges facing the cities? For instance, could the regeneration be regarded as a type of decontextualisation, and a way of ensuring opportunities for natural-based recreation in a dense urban environment? How could the regeneration of urban water infrastructure be viewed as aiming at, as Davies says, “revitalizing an area economically, socially, environmentally, and physically”? How could the regeneration of urban water infrastructure be related to the concept of ecosystem services? What seem to be prerequisites for successfully incorporate these perspectives when developing the urban water infrastructure?

## 3 Materials and methods

As mentioned in the introduction, this paper aims at exploring the underlying factors behind, and the potential benefits and challenges of, recreation- and sport-led regeneration of urban water infrastructure, with a focus on former harbours and canals. The study is framed by the concepts and the theoretical background presented above. Empirically, the development of water infrastructure for swimming and other water activities in urban canals and harbours, and different perspectives on this development, will be illustrated and discussed based on the example cities. The cities focused on in this study have had extensive harbour areas and used to have ship-building industries. During the last decades, the cities have worked with regeneration projects in the former industrial harbour areas. The three selected Scandinavian cities share similar planning systems, characterised by strong municipal/public authority. They vary in size, ranging from a large capital city to a medium-sized regional centre. Additionally, they have been selected because they represent different solutions and stages in harbour development for recreational purposes. Thus, they exhibit both similarities and interesting differences. The examples used in this paper are:

1. Copenhagen, with a focus on the comprehensive and extensive development of urban swimming opportunities in canals and in the harbour area.
2. Malmö, with a focus on the development of the waterfront, especially the old wharf and harbour area, and the future plans for the canal.
3. Gothenburg, with a special emphasis on the harbour swimming area (Hamnbadet) in the Jubileum Park.

The study has a qualitative approach. Insights about the example cities have been collected through interviews with experts of each case, supplemented by written documents (like municipal plans, programmes and visions). The experts represent the institutions responsible for the recreation- and sport-led regeneration of urban water infrastructure, which, in all three cases, is the municipality. The municipal experts are civil servants in leading positions within municipal departments. In one case (Gothenburg), a non-profit organisation (Passalen) is responsible for the activation of the place in question, alongside the municipality. Therefore, a representative from Passalen was included among the informants.

The interviews had a semi-structured format. To capture the key aspects of the study's aim, the interview guide was structured around the following overarching questions:

1. Describe your role in the development of urban water infrastructure and urban recreation.
2. Describe the city's water infrastructure and its development over time.
3. Explain the significance of water infrastructure to the city's identity.
4. Discuss the importance of water infrastructure for the city's development.
5. Describe the relationship between the water infrastructure and the city's residents.
6. Explain the role of water infrastructure in engaging and activating the city's residents and visitors.
7. Reflect on the development/regeneration of former harbour areas and canals, considering:
  • Values
  • Objectives
  • Target groups
8. Elaborate on the driving forces and ambitions, as well as the effects, of recreation/sport-led regeneration of urban water infrastructure.
9. Identify the challenges and problems associated with recreation/sport-led regeneration of urban water infrastructure.
10. Relate the development of harbour areas/canals to the following perspectives:
  • Creating activity opportunities for residents in public spaces
  • Urban regeneration, including the renewal of industrial and harbour areas
  • Attracting tourists and building an urban brand
  • Pursuing sustainable development

The sequence of questions may vary depending on the interview's flow. Follow-up questions were used to delve deeper into specific themes or responses and to capture local variations. For instance, one of the interviews (with Ia in Gothenburg) partly went in another direction content-wise, as the informant had a different role than the rest of the informants.

The interviews were conducted in October and November 2024. Each interview lasted 65–88 min. Three individual interviews and one group interview, with altogether six informants, were conducted. The individual interviews all took place on Zoom or Teams, while the group interview had a hybrid format with two informants joining online. All interviews were recorded and transcribed. Based on the key aspects of the study's aim, a step-wise content analysis was performed, grouping the interviewees' responses into categories and sub-categories. The results are presented mainly in the form of statements and exact quotes, mixed with input from different documents.

The informants are listed below, with correct first names and roles/titles but no surnames. All informants have received sufficient information about the study and their rights and have signed an informed consent form. None of them have requested anonymity.

Informants:

• Lars: chief advisor, Culture and Leisure Department, City of Copenhagen.
• Agneta: head of the urban environment unit, Streets, Parks and Property Department, City of Malmö.
• Pella: project leader, unit of public environment, Streets, Parks and Property Department, City of Malmö.
• Peter: strategist, unit of public environment, Streets, Parks and Property Department, City of Malmö.
• Johan: head of department, Urban Environment Department, City of Gothenburg.
• Ia: operation manager, the non-profit association Passalen, Gothenburg.

To do the study justice, it is important to underline that the aim is not to measure the outcomes of sport- and recreation-led regeneration of urban water infrastructure; the aim is instead to explore the factors and motives behind it, by reflecting on different perspectives and, in relation to this, on potential outcomes. To fulfil this aim, the study has a producer rather than a consumer focus, the informants being involved in the planning, production and operation of the water infrastructure. Consequently, the users are not given a voice in this study. Relying solely on the perspective of the producers carries the risk of presenting an uncritical description of the development. As the interviewer, I had to take this into consideration when conducting and analysing the interview material. If continuing with a study on the outcomes, a consumer focus would be useful.

The aim of the cases/examples, and not least the voices of the informants, is not to present identical material in order to compare the cases, but to show how different perspectives come into play in relation to sport- and recreation-led regeneration of urban water infrastructure in different settings. Hence, little focus is placed on presenting the three cities in detail.

## 4 Three urban examples

### 4.1 Copenhagen: outstanding harbour swimming and recreation

The Danish capital Copenhagen, with approximately 1.4 million inhabitants in the Greater Copenhagen region, is located by the Öresund (the sound between Denmark and Sweden). The city has beaches along the sound outside the inner city and extensive former harbour areas as well as canals in the inner city. Today, as stated by the informant Lars, chief advisor at the Culture and Leisure Department, the city is one of the best cities in the world for harbour swimming and recreation, which has been noticed in international media as well, for example, in an article in the New York Times: “Copenhagen's harbor was once polluted with sewage, industrial waste and oil spills. But the Danish capital has slowly evolved into one of the best cities for year-round swimmers” (1, no page).

In the 1990s, the city started to clean up the harbour and the canals and transform them into recreational spaces (57). As part of this, the city invested in modernising its sewage system, rerouting wastewater into underground basins and expanding wastewater treatment plants. It was very costly but, as a result, the water in the harbour and the canals is clean and biodiversity has improved considerably.

The harbour regeneration concept in Copenhagen builds on recreational use of the harbour and accessibility for everyone, Lars emphasises. Already some 30 years ago a promenade policy was adopted. It stated that when regenerating the harbour areas, an 8–12-metre-wide public promenade strip must be saved along the water. This rule applies regardless of who owns the land. The result is that 95% of the area offers access to a promenade strip (for, e.g., walking, biking or just sitting) along the water. “It's attractive for the residents and a nice way to experience Copenhagen”, Lars says and then adds: “also, it has affected the housing prices positively along the water”. The development has involved not only traditionally attractive areas, but also industrial areas. Lars highlights different activities that are enabled through the regeneration of the areas along the canals and the harbour: it has become possible to see, come close to and walk by the water, and to dip one's toes in it and swim.

An important step in formalising the process of regenerating the harbour was the adoption of a vision for the harbour, called “En havn af muligheder” (in English “A harbour of opportunities”) (58). The vision is based on eight themes which should guide the development of the harbour area (see p. 13): a variety of activities; better access to and from the water; more public places; improved routes and connections; a clean and attractive harbour; a harbour with a healthy environment; events and temporary activities; and variation and space for everyone.

Although Copenhagen has become well known for having one of the best harbours, or *the* best harbour, in the world to swim in, the harbour is still a busy waterway and swimming is only permitted inside designated bathing zones. There are 16 such zones of different kinds, all available for free:

• Three harbour baths (pool areas), which are large facilities with a high degree of security and comfort, and with an attractive design. They contain pools of different sizes and depths with nets at the bottom, and piers and diving towers. Lifeguards survey the facilities during the summer months. For examples, see, e.g., Visit Copenhagen (59).
• Ten bathing/swimming zones, which are a bit simpler than the harbour baths, more like urban concrete beaches. The zones are marked, they do not contain any nets at the bottom, and there are no lifeguards.
• Three dip zones, which are safe places for getting into the water.

Lars describes the advanced system for continuously measuring and monitoring the water quality (in real time). At all the 16 bathing zones, there is a screen showing if the water quality is red or green, or you can see it on your mobile phone. It is almost always green.

Lars contends that the main challenge for further developing the harbour area is the competition for space in the water. The swimmers like to have the harbour for swimming, while the harbour buses (public transport on water) and the canal boats also want to use the water. Water activities, like stand-up paddle boards (SUPs), rowing and kayaking, desire space as well. Moreover, the harbour and the canals are very attractive for different types of water-related events. A favourable factor is the good access also for spectators on the quaysides, thanks to the promenade strip mentioned above. A growing number of events related to the water and organised by commercial actors as well as the municipality and sport clubs and associations, are competing for time and space. The events cover everything from smaller community-oriented events to large, international events, like the 2024 ISA World SUP & Paddleboard Championship, and spectacular, commercial events like the Red Bull Cliff Diving from the opera house. A popular annual event is the TrygFonden Copenhagen Swim, where the participants swim in the canals around the Parliament. “And everyone who can swim can join the event! We have participants from the age of 7–82, we have war veterans, handicapped swimmers, beginners and elite swimmers making the 2,000 m swim around the Parliament – either alone or in teams” (60, no page).

Besides the challenge of the competition for space in the water, there is a challenge with regard to different ownerships on land. Some of the land along the canals and the harbour is publicly owned, by the state, the municipality or the public-public partnership By og Havn, and some is privately owned. The rule to make a strip along the water available for everyone applies to all landowners, which sometimes creates a bit of dissatisfaction among the residents in a specific area when people from outside use their quayside for recreation and socialising. Also, in some cases several different landowners are present within a limited area, which can make it difficult to sort out the responsibilities. Yet another challenge concerns the illegal swimming outside the dedicated swimming areas, which is a security risk in the busy waters. However, Lars notes that accidents are very rare.

The harbour swimming areas attract a lot of the Copenhageners and a growing number of visitors. According to Santamaria (57, no page), “Copenhagen Harbour has become a key attraction in its own right”. However, as Lars states, “swimming in Scandinavia is often a fairly primitive activity”. This does not necessarily attract people from other cultures and countries. Therefore, the next step is to develop swimming areas with a higher degree of comfort, containing, for instance, dressing rooms, showers and toilets. This could be viewed as developing the community-oriented approach towards a more tourist-oriented approach.

### 4.2 Malmö: much water and many challenges

Malmö is the third largest city in Sweden with approximately 365,000 inhabitants. By Swedish standards, Malmö is considered to be a segregated city, with differences in, for example, health status, physical activity levels and sport participation between different population groups. The city has a strong industrial past, among other things based on ship building. Located by the Öresund coast, just like Copenhagen, Malmö used to have extensive port areas, most of which have today lost their former use. Some of the former harbour areas have been transformed/regenerated and contain residential areas, university buildings and offices. Other areas are waiting for regeneration. Furthermore, the historic city centre is surrounded by a six- kilometre canal, which was built in the early 19th century based on old water graves. These connect to the three different parts of the harbour (see, e.g., 61).

During the interview with the informants at the Streets, Parks and Property Department, the strategist Peter starts by reflecting on the role of water in and for Malmö. During the last 20 years, the water has taken on a new meaning for the city. Except for the beaches, the connection to water used to have an industrial role. When the area called Västra Hamnen (the Western Harbour) started to be developed and transformed into a residential area, the role of and the access to water changed for the residents of Malmö with new forms of recreation and outdoor environments, and, more excitingly, urban swimming opportunities. “The city centre has approached the sea, city life is now connected to the sea. We are in the middle of a big change. This will be even more clear in the former industrial harbour areas being developed right now, like Nyhamnen (New Harbour)”, Peter says.

The project leader Pella's first reflection is that Malmö has unique opportunities for urban beach activities and swimming, compared to some other coastal cities, like Gothenburg. Despite this, she continues, Malmö is not perceived as a city by the water and rarely mentioned as such in the context of tourism. Tourism had not been in focus when planning and developing the water-related areas. Even some of the residents in Malmö are not aware of the opportunities that the coast and the water bring, according to Pella. Agneta, head of the unit, points out that this is partly because the water is concentrated to the western parts of the city, which are the wealthy areas. She adds that one of the ambitions is to create accessibility for more people to come close to the water.

The informants highlight that the coast and quays could be considered a joint living room for the residents, although a lot of people do not use them today. However, the potential lies in the fact that they are quite a neutral space, and not programmed for a certain activity.

In the Master plan for Malmö (62), one of the prioritised strategic areas regards green and blue environments. In the plan it is, among other things, stated that

• Urban development must contribute to Öresund becoming a healthy and living sea with a rich plant and animal life, where opportunities for recreation and experiences are combined with fishing, shipping and energy production.
• Accessibility to the coast, waterways, canal, harbour basins and quaysides for recreation and leisure activities must be improved. A continuous coastal strip must be created along the municipality's coast.
• The City of Malmö must actively work to ensure the ocean's ecosystem services in the long term.

Along the sea, the city has long sand and grass beaches, some of them in close proximity to the inner city. These are a huge asset in the city, also complemented with recreational areas on land. Moreover, Malmö, just like many other cities, has developed into an event city. Often, the fact that Malmö hosted the Louis Vuitton Acts 6 & 7 of the 32nd America's Cup in 2005, is considered to mark the beginning of this development. Since then, Malmö has organised many events by the sea. The sea is used not only as a direct resource but also as a backdrop for non-aquatic events. One such event is the Malmö City Horse Show and another is Toughest Malmö (63).

In this paper, however, the focus is on the development of both former industrial/harbour and canal areas for water activities.

When developing the Western Harbour area at the turn of the century (the first residential areas opening in 2001), swimming was not allowed. However, many people gathered along the promenade and on the lawns in the summer, and many swam in the sea despite the ban. Hence, to meet the the obvious needs for swimming facilities, in 2006 the City of Malmö established two places for deepwater swimming: at the promenade and further out in connection to a recreational area, and they were equipped with decks, piers, stairs and toilets, as well as lifeguards during the summertime. Both places face the open sea, and not the docks and inner ports. They are very popular, especially among teenagers, and the residents in the area often express concerns with the crowds, noise and littering. In other parts of the Western Harbour, swimming is not allowed, but activities like kayaking take place and one of the docks functions as a yacht harbour.

There are discussions regarding the development of the canal in the inner city and the possibility of turning it into a space for more recreation activities. Agneta points out that “politicians like to state that in year X you can swim in the canal, but we, as a municipal department, do not own that process. For us, it is not worth starting to plan [for that] yet if the water isn't clean”. Peter emphasises that purifying the water is a long, expensive process.

Also, having an extensive beach makes it less interesting, or necessary, to develop the canal and make it safe for swimming. It is nevertheless worth improving the water quality to make the water infrastructure safe for recreational transports, like kayaking, Agneta thinks. Already today, the canal is used for boat-related activities and for sport fishing. One of the users is Malmö Canoeing Club, whose club house is beautifully located with direct access to the water.

As part of the ambitions to develop the canal in the future, the City of Malmö commissioned an architectural firm to present a vision for the canal. The report “Malmö kanalrum” (in English, Canal spaces of Malmö) (61) aims to show how Malmö can come closer to the water and raise the canal space to become a stronger part of the city's identity. The report builds on a programme for developing the canal from 2014. Since then, several places along the canal have been developed to make it more approachable and accessible. However, the canal is still in need of renovation and the water is heavily polluted. Ideas for a step-wise development of the canal include making it more accessible for recreation by building promenades, stairs, piers, pontoons, etc., and, in the long run, suitable for swimming—both in separate pools with clean water and in the canal itself. The time frame for the latter might be as long as 25–30 years.

Where the canal flows into the harbour basin the water quality is not good enough for swimming and other water activities, Peter underlines. Despite this, competitions in water sports like water polo were held in the harbour basin during the Swedish championships in 2019. However, the participants had to sign an agreement to compete in the polluted water!

Malmö is in the process of further developing quaysides and access to water. In December 2023, the result of an investigation carried out by a consultant firm was presented in the report “Activating the quays in central Malmö” (64). The aim was to create an understanding of “the conditions and circumstances for how quays and the port area can be activated and what opportunities and obstacles there are to succeed in doing so” (p. 3).

The investigation highlights two main obstacles for water activation in the harbour area. The first is the port ordinance, which does not allow an official place for swimming to be established in the old docklands. The second regards the fact that the pollutions in the water are too high. A new sewer tunnel is being built, but it will not eliminate the problems with polluted storm water. A third complicating factor is the extensive depth of the water basins/docks, as it used to be an industrial port and was used for, among other things, the launching of submarines.

According to the report, there is a risk that people are tempted to swim when the quaysides are developed and provided with walks, stairs and piers. One way of solving the issue is to build separate pools with clean water that are submerged in the harbour basin. However, this is a very costly solution and does not ensure that people do not swim outside the pools.

Pella expresses the ambition of the work with the quaysides like this: “It is about creating opportunities to let water activities meet land activities”. However, the water quality in the canal and in the area where the canal flows into the harbour basin, is a limiting factor, something the informants repeatedly come back to during the interview.

### 4.3 Gothenburg: community-led development of swimming in the harbour

Just like Malmö and Copenhagen, Gothenburg is a previously industrial city with extensive harbour areas. It is the third largest city in Sweden with approximately 608,000 inhabitants in the city and just above 1 million in the metropolitan area (65). The western, outer parts of the city are connected to the sea, with a rich archipelago, while inner parts stretch along the river Göta älv, which runs into the sea outside the city. The more industrial parts of the harbour are mainly concentrated to the river, which is polluted and thus not suitable for swimming. It is only during the last decades that the urban development has started to turn towards the river, as the old harbour areas are undergoing a regeneration. Similar to Malmö, Gothenburg is a fairly segregated city, from a Swedish perspective, and access to water for recreation is segregated as well.

The Gothenburg example will focus on the only swimmable spot in the harbour: Hamnbadet (the Harbour Bath), and the interesting approach behind it. Johan at the Urban Environment Department describes how the development of Hamnbadet and Jubileumsparken (the Jubileum Park—a park connected to Hamnbadet) is based on a far-reaching participation of the residents in Gothenburg. When the City of Gothenburg planned for its 400th anniversary, the city had a dialogue with the residents to obtain ideas and wishes for the urban development. A lot of the wishes were connected to two themes: a greener city and getting closer to the water in the inner city, including being able to swim there. These two main desires formed the basis when developing Hamnbadet and Jubileumsparken in the former industrial harbour area.

The area where Hamnbadet and Jubileumsparken are situated, which is described by Ia at Passalen as having been a rough, hard surface unsuitable for recreation, was used as a test bed or living lab for ideas developing over a 10-year period. Residents, mainly children and young people, also from minority groups, participated in the process. Their desire for a greener city and the possibility to swim in the inner city were translated into full-scale prototypes. In the water, different types of pools were constructed, and on the pier a prototype sauna with a spectacular design was built. Residents helped testing and evaluating the different prototypes. It was an explorative and sometimes bumpy road, Johan emphasises during the interview. Based on this process, permanent solutions were developed. The process and the inclusion of the residents made the park and the harbour bath well known and popular already during the planning phase.

When finalised in 2023, Hamnbadet contained a saltwater exercise pool and a jumping pool with a trampoline, both open all year, as well as a freshwater play pool with a bottom. The pools are surrounded by nice piers and stands, and there is an eye-catching sauna with a harbour view. Everything is free. Outside the pool area, in the harbour, sailing and SUP activities are offered, despite the poor water quality.

The park and the bath have been integrated. For instance, sand filters in the park are used for cleaning the pool water. No chemicals are used. Johan states that several ecological system services are contributed by the area. No one owns the land, as it is so-called neutral land, Johan explains. The location in the city is also neutral, not signalling any specific status. The City of Gothenburg shares the responsibility for Hamnbadet with the non-profit association Passalen. Ia, the operation manager of Passalen, describes it as a children's rights organisation focusing on an active life and inclusive leisure for all children, also those with disabilities. Passalen takes care of the operations and activities, with public support, while the city handles the technical issues.

Passalen provides a large range of activities at Hamnbadet, not only for children. The pool area is open for spontaneous use, but Passalen also organises, for instance, morning swim programmes for adults and seniors, and swim training for people who are new to Sweden. Outside of the pool area, canoeing, SUP and sailing are organised. There are, furthermore, summer camps for people with and without disabilities; a sailing school; school activities; and outdoor classrooms where children can learn about water. Ia underlines that water access and activities are often associated with Swedish middle and upper classes. However, by having an inclusive approach, the activities at Hamnbadet aim at introducing, for instance, sailing to a group that is not the usual target group for water activities.

The aim is to be a location for all population groups of all ages, from Gothenburg and elsewhere both inside and outside of Sweden. And the bath attracts a variety of people. However, visitor statistics collected during 8 months in 2024 shows that the most common visitors are adults and children, despite the special focus on young people. The majority, 56%, are males and 44% are females. Around 50% of the visitors are from other Swedish places or from abroad (66). This proves that Hamnbadet has developed into a popular tourist attraction.

Hamnbadet and Jubileumsparken are not an event venue, but a mini-triathlon for all ages and abilities opened the summer season 2024. The event was a way to show the inclusive approach and attract people to the location (67).

The development of Hamnbadet and Jubileumsparken, although being a unique project, fits well with the ambitions in the Plan for urban outdoor recreation, adopted by the City of Gothenburg (68):

The goal of urban outdoor life is for as many people as possible to, in a simple and easily accessible way, be able to use their local environment for recreation. By facilitating physical activity in the urban environment, more target groups can be reached and contribute to the city's overall goals, if increased public health and a more equal Gothenburg are reached at the same time as the urban environment is experienced as more attractive (p. 4).

Partly due to the poor water quality in the harbour, the City of Gothenburg has no stated strategy to open up more spots for water activity. Rather, they will focus on recreation on land with water contact, like attractive quays and a greener encounter with the water.

As described, the concept behind Hamnbadet is very community focused. However, the unique and interesting design has attracted a lot of attention from both tourists and planners around the world. When people arrive at the airport in Gothenburg, they are welcomed by big pictures of Hamnbadet. And the sauna, with tall harbour cranes in the background, adorns the cover of the photographic anniversary book published when Gothenburg was celebrating 400 years.

Ia summarises the core of the concept of Hamnbadet, in describing the cooperation between the City of Gothenburg, the back-bone, and Passalen, the facilitator for inclusion and the adopter of a norm-critical perspective: “Together, we have created a free, accessible, universal design and concept, where everyone is a VIP”. She also underlines the “wow factor” of the facilities, not least the sauna.

## 5 Summarising discussion

The three examples above are used to explore the underlying factors behind, and the potential benefits and challenges of, a recreation- and sport-led regeneration of urban water infrastructure with a focus on former harbours and canals. They have showcased different prerequisites, opportunities, benefits and obstacles in the process of regeneration of urban water infrastructure in former industrial cities.

The example cities represent different stages in the process, Copenhagen being by far the most well-developed example with a 30-year-long history of successfully cleaning and making the harbour area suitable for water activities. The first so-called harbour bath in Copenhagen opened in 2002 (69). In Malmö, the harbour regeneration has been going on for more than 20 years. However, it has mainly focused on the parts facing the sea (the Öresund). The planning for developing the canal and the inner harbour areas, and transforming them into clean recreational spaces, has been ongoing for about 10 years. Investigations and visions have been developed, but no concrete actions have been taken, except for renewing some of the access points to the canal. Here, the “where land meets water” approach is important. In Gothenburg, by contrast, there are no comprehensive plans and visions for developing the harbour water areas into recreational areas, except for the specific case presented in this paper: Hamnbadet. Hamnbadet is to be viewed as a test bed for participatory planning based on the desires of the residents to be able to swim in the inner city. This highlights a very central perspective of recreation-led regeneration of urban water infrastructure, namely, the focus on the residents and the needs of the community. The three examples have one aspect in common: an emphasis on bringing the water closer to the residents and making recreation in the former harbour areas possible. This type of development could also be regarded as a response to the growing preferences for self-organised activities (e.g., 25). None of the examples emphasises the tourism perspective as a driving force, which is very interesting as sport-related urban regeneration often has been viewed from a city branding and tourism perspective. Still, all the example cities, not least Copenhagen, have experienced different types of branding and tourism effects related to the regenerated water infrastructure. Tourists and events are attracted to the regenerated harbour areas. This shows that community-oriented regeneration has the potential to create both branding effects and attractive, functional opportunities for the residents. Today, many tourists are said to aim for more genuine, local experiences, besides or instead of the more traditional tourist attractions (for a discussion on authenticity within the context of tourism, see, e.g., 70). This could, in turn, be linked to the growing interest in so-called slow tourism, which refers to a type of tourism in which the tourists take their time and engage with people and places. “Consistent with these forms of tourism, the slow idea achieves sustainable development through authentic experiences” (71, p. 397–398). Recreation-led harbour regeneration gains the interest not only of users, that is, both people living in the city and tourists, but also of experts in other cities. As stated by Jensen et al. (69, p. 555): “Today, the harbour baths are associated with the discourse of the green liveable city, which has attracted the interest of many urban planners and architects”. From a sport and recreation perspective, the concept of “sport urbanism” could be applied here as well. Moreover, the holistic approaches to waterfront redevelopment, highlighted by Tommarchi (9) could also be applied in relation to the three examples. In this context, Sairinen and Kumpulainen (12) discuss emerging ideas of regeneration with broader concepts of environmental sustainability, including the social dimension and community targets.

The approaches in Copenhagen and Gothenburg differ, despite focusing on the same thing: creating access to water and swimming. In Copenhagen, the harbour development has been a comprehensive, conscious strategy, well-grounded in visions and plans, and representing more of a top-down perspective. Landowners along the canals and harbour water ways have been forced to adapt to the strategy, which in turn has created value for everyone—from the general public to the land and estate owners. The overall idea seems to be to create attractive urban development. Although the harbour development started at the top, “today, swimming in the harbour has become an institutionalised urban practice” (69, p. 554). In Gothenburg, Hamnbadet has been developed in a more explorative way based on a high degree of community participation, that is, a bottom-up approach. Now, when finished, Hamnbadet is run according to a public-nonprofit partnership concept with an idea of creating social capital (for a discussion, see 72). However, the “wow factor” has gained the urban development as well and brought with it some positive economic consequences.

As shown in the examples, recreation-led regeneration of urban water infrastructure comes with several benefits. However, a number of challenges and obstacles are illustrated by the three examples. The most obvious obstacle has to do with the water quality. Harbour areas and canals are often highly polluted. To clean the water, and monitor the quality—as is done in Copenhagen—is a complicated and costly process. But once the water is cleaned, biodiversity and recreational values increase.

Another challenge has to do with providing equal access to the water infrastructure. Water access and activities are often associated with higher socio-economic status, creating barriers for a lot of residents. As presented above, previous studies have shed light on accessibility aspects in connection to (public) places for sport and recreation (see e.g., 31, 32, 37). The example cities have worked with and plan to adopt different types of approaches for giving access to water, focusing both on the edges where water meets land and on the different activity opportunities in the water. Moreover, services and supporting infrastructure, such as decks, piers, toilets and lifeguards, would increase the comfort and usability for more people. In the case of Hamnbadet, special attention has been paid to social support, representation and the feeling of belonging.

To develop former industrial spaces—in this case, water infrastructure—into recreational areas, which could be regarded as some kind of public sport facilities, is a smart way of finding new spaces for development in growing and dense urban areas. In many cities undergoing densification, there is a discussion around the lack of green spaces (see, e.g., 50). In this perspective, although blue spaces cannot replace green spaces, they can have a compensatory effect and contribute with values, such as ecosystem services (see, e.g., 73). However, when making the water infrastructure suitable for recreation, competition over space in the water increases. Hence, the competition over space and the densification that we have seen on land in many cities, is something that we are now witnessing in the water as well. Boats, canoes, swimmers, people fishing, SUPs, pools, piers, residents, tourists and so on, must co-exist in the same space. Sometimes, events are added to the equation as well. In Copenhagen, for example, the harbour waterways are very busy, which is also a safety risk. In relation to this, another challenge is to prevent people from swimming outside dedicated swimming areas.

Yet another challenge when opening up the harbour areas for recreation for the general public, is the different ownerships and responsibilities on land. In the case of Copenhagen, rules for saving a public promenade strip along the water create access for everyone but also potential conflicts. Moreover, in the water there are different actors with different agendas involved. Although the harbour development in Copenhagen is described as a success story, the road forward has not always been straight. As described by Jensen et al. (69), it has involved navigational actions between different actors, interests, enactments and visions.

In the case of Malmö, there are also regulations working against activating the harbour, namely, the port ordinance, which does not allow an official place for swimming to be established in the old docklands.

If considering all the benefits, for instance in terms of ecosystem services, such as enhanced biodiversity and improved recreational opportunities, quality of life and well-being (e.g., 52), the positive long-term effects could (or should) outweigh the extensive cost for cleaning and developing the harbour and the canals. The economic benefits in terms of tourism and positive attention could also be significant. However, where there is already a large supply of beaches near the inner city, as in Malmö, the driving forces for developing the harbour areas and the canals seem to be weaker. The added value might be smaller than if there are no swimming opportunities nearby.

When regenerating the harbour, water takes on a new meaning: from industrial to recreational purposes. So, how could swimming and, for instance, kayaking in the harbour basin be interpreted, using the active domination approach and the active adaptation approach, as presented by Sandell and Öhman (44)? Are the activities adapted to the new recreation landscape, or is the landscape being manufactured, structured and equipped for certain activities? Probably both, as it is a two-way negotiation and decontextualisation process, to use Sandell's (45) concept. It is a way of finding new spatial solutions as well as urbanising recreational activities, formerly performed in natural settings.

## 6 Concluding words

As observed in the three example cities, swimming is just one of many recreational water activities associated with the development of urban water infrastructure. Nevertheless, urban swimming is a significant component, attracting growing interest and facilitating knowledge exchange (74, no page):

Launched in the lead up to the Paris Olympics in July 2024, the Swimmable Cities alliance is supporting a global, grassroots movement for swimmable urban waterways. With 100 diverse signatory organisations from 59 cities and communities and 22 countries, our Swimmable Cities Charter champions the Right to Swim, celebrates urban swimming culture, and honours the sacredness of water.

The Paris example at the beginning of this paper spured my interest for conducting a study concerning sport-related use of harbours and canals. The three Scandinavian cities were used as illustrative examples for exploring recreation- and sport-led regeneration of urban water infrastructure. Although, the Seine example is to be considered as both interesting and problematic and the Copenhagen example as unusually successful, these are just examples among many potential candidates. There is a growing number of (mainly larger) cities in different parts of the world gaining attention for their work with recreation- and sport-led regeneration of urban water infrastructure. For instance, Oslo, Berlin, London, Munich, Basel, Vienna, Amsterdam, Baltimore, Sydney, etc. (see, e.g., 1, 75). However, it would be interesting to also learn from smaller cities. I believe that the experiences from the three example cities can be applied to other geographical contexts as well, either as comprehensive approaches as in Copenhagen or more limited approaches focusing on a specific place like in Gothenburg.

**Figure 1:**
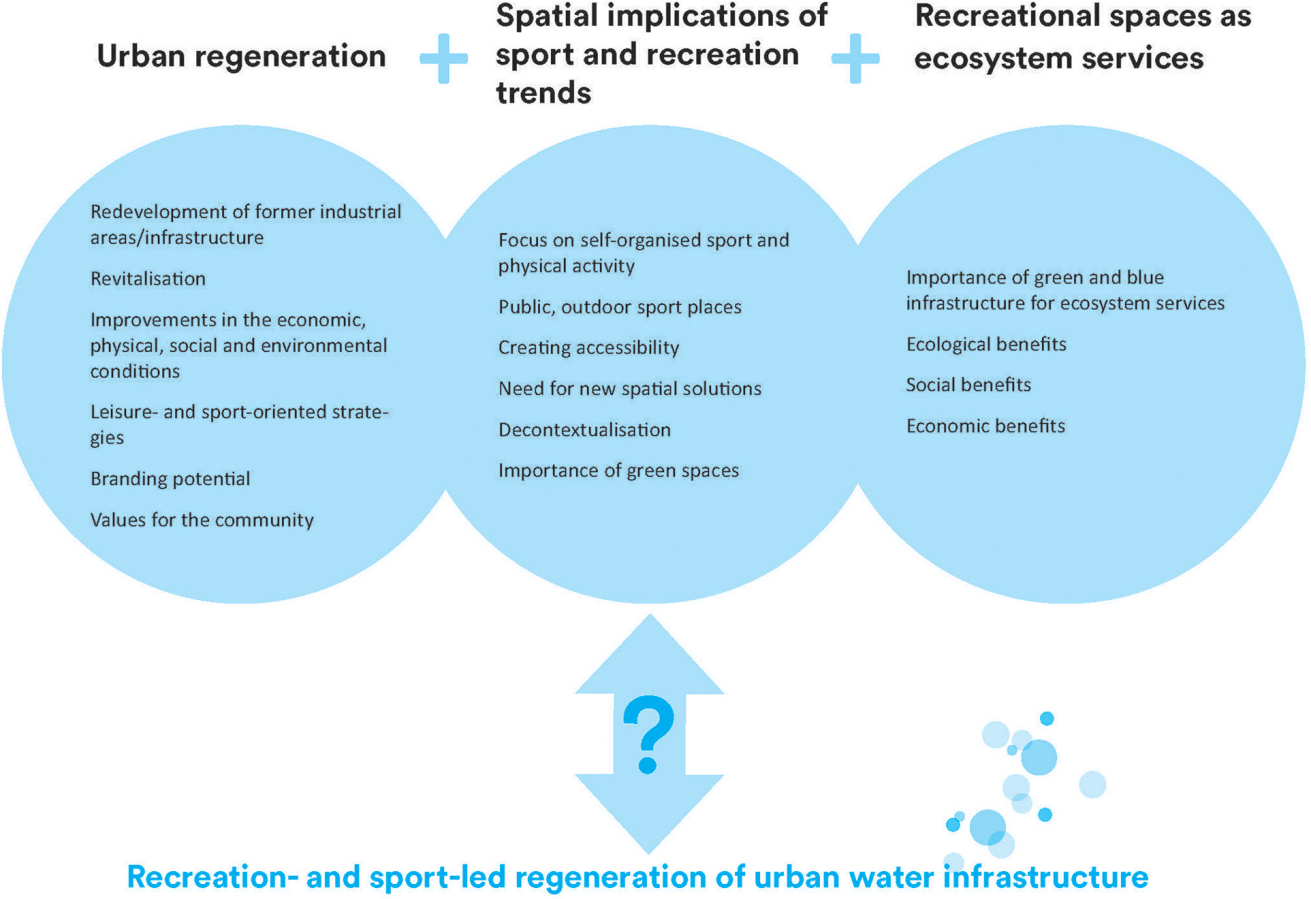
Conceptual framework for interpreting the growing phenomenon of recreation- and sport-led regeneration of urban water infrastructure.

As shown in Figure 1, the recreation- and sport-led regeneration of urban water infrastructure can be connected, to varying degrees, to the following overarching perspectives: urban regeneration, spatial implications of sport and recreation trends, and ecosystem services. These perspectives are present in the three examples in this paper.

When examining the underlying factors behind the (plans for) regeneration of the harbors and canals in the cities, it becomes evident that, contrary to what is usually the case in large-scale waterfront (re)development (e.g., 5), branding and tourism are not the main driving forces. Instead, the needs of the residents and the community, a response to the growing preferences for self-organized activities, and the discourse of the green, livable city seem to be the guiding principles.

The examples highlight several benefits of a recreation- and sport-led regeneration of former harbors and canals, including enhanced biodiversity, improved recreational opportunities, increased quality of life and well-being, economic benefits in terms of tourism, positive attention and value for land/estate owners, and opportunities for participatory planning processes.

As the paper demonstrates, using three example cities, successful regeneration of harbours and canals can lead to increased attractivity, activity and sustainability. However, the extent of the benefits, particularly those related to environmental sustainability, is closely tied to the scale of the canal and harbour renewal.

**Figure 2:**
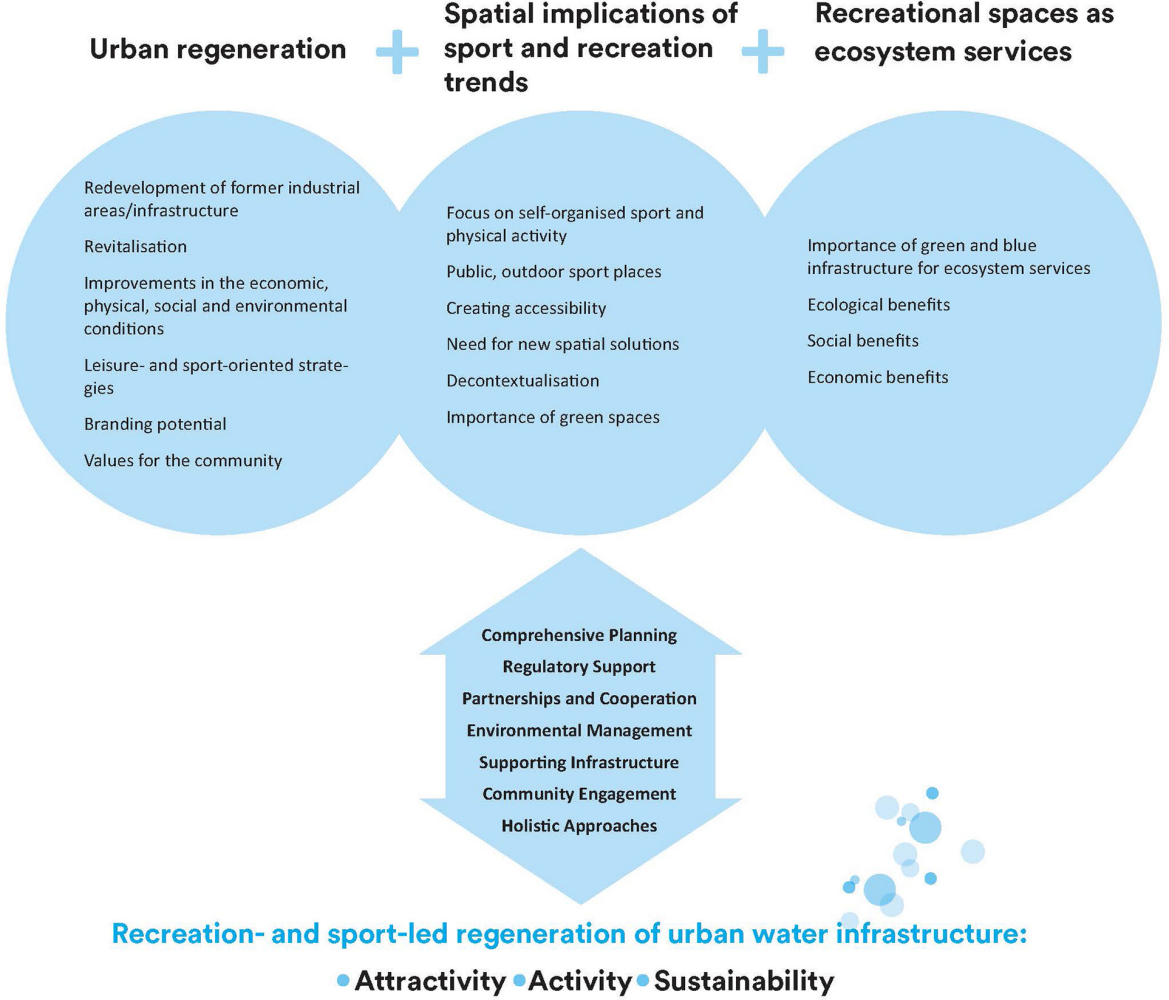
Perspectives and prerequisites for recreation- and sport-led regeneration of urban water infrastructure.

The empirical examples also provide insights into the prerequisites for successful regeneration of harbours and canals for recreational purposes. These are related to the challenges identified in the examples, and the ability to address them. The prerequisites, as shown in Figure 2, include:

• Comprehensive Planning: Developing a clear, strategic plan that integrates recreation, sport, and environmental goals.
• Regulatory Support: Establishing regulations that facilitate access and sustainable use of water infrastructure.
• Partnerships and Cooperation: Building strong collaborations between government, private sector, and community organisations.
• Environmental Management: Ensuring effective cleaning and ongoing monitoring of water quality.
• Supporting Infrastructure: Providing necessary services and infrastructure, including social support systems.
• Community Engagement: Involving residents in the planning process to ensure their needs and preferences are met.
• Holistic Approaches: Applying comprehensive redevelopment strategies that consider multiple aspects of urban living.

## Data Availability

The datasets presented in this article are not readily available because those interested in the data are welcome to contact me to discuss and get more information about the data. Requests to access the datasets should be directed to karin.book@mau.se.
